# PREPARE: protocol for a stepped wedge trial to evaluate whether a risk stratification model can reduce preterm deliveries among women with suspected or confirmed preterm pre-eclampsia

**DOI:** 10.1186/s12884-019-2445-x

**Published:** 2019-10-07

**Authors:** Marcos Augusto Bastos Dias, Leandro De Oliveira, Arundhanthi Jeyabalan, Beth Payne, Christopher W. Redman, Laura Magee, Lucilla Poston, Lucy Chappell, Paul Seed, Peter von Dadelszen, James Michael Roberts, PREPARE Research Group, Maria Laura Costa do Nascimento, José Paulo Guida, Carla Silveira, José Guilherme Cecatti, Francisco Lázaro Pereira de Souza, José Geraldo Ramos, Sérgio Martins Costa, Marcos Antonio Santos, Guilherme de Jesus, Fátima Penso, Lucienne Frayne, Wallace Mendes, Marcos Nakamura, Lucienne Frayha

**Affiliations:** 1 Fernandes Figueira Institute, Rio de Janeiro, Brazil; 2 Medical School, Obstetrics Department, Botucatu Sao Paulo State University, Botucatu, Brazil; 3 Department of Obstetrics and Gynecology, University of Pittsburgh, Pittsburgh, PA USA; 4 Healthy Starts Theme, BC Children’s Hospital Research, Vancouver, BC Canada; 5 Department of Anaesthesiology, Pharmacology and Therapeutics, University of British Columbia, Vancouver, BC Canada; 6 Nuffield Department of Obstetrics and Gynaecology, University of Oxford, Oxford, UK; 7 Department of Women and Children’s Health, School of Life Course Sciences, King’s College London, London, UK; 8 Division of Women’s Health, Women’s Health Academic Centre, King’s College London and King’s Health Partners, St. Thomas’ Hospital, 10th floor, North Wing, London, UK; 9 St. George’s, Hospitals NHS Foundation Trust, University of London, London, UK

**Keywords:** Pre-eclampsia, Preterm birth, Prematurity, sFlt-1/PlGF, fullPIERS

## Abstract

**Background:**

Preeclampsia (PE) is a major cause of short and long-term morbidity for affected infants, including consequences of fetal growth restriction and iatrogenic prematurity. In Brazil, this is a special problem as PE accounts for 18% of preterm births (PTB). In the PREPARE (Prematurity REduction by Pre-eclampsia cARE) study, we will test a novel system of integrated care based on risk stratification and knowledge transfer, to safely reduce PTB.

**Methods:**

This is a stepped wedge cluster randomised trial that will include women with suspected or confirmed PE between 20 + 0 and 36 + 6 gestational weeks. All pregnant women presenting with these findings at seven tertiary centres in geographically dispersed sites, throughout Brazil, will be considered eligible and evaluated in terms of risk stratification at admission. At randomly allocated time points, sites will transition to risk stratification performed according to sFlt-1/PlGF (Roche Diagnostics) measurement and fullPIERS score with both results will be revealed to care providers. The healthcare providers of women stratified as low risk for adverse outcomes (sFlt-1/PlGF ≤38 AND fullPIERS< 10% risk) will receive the recommendation to defer delivery. sFlt-1/PlGF will be repeated once and fullPIERS score twice a week. Rates of prematurity due to preeclampsia before and after the intervention will be compared. Additionally, providers will receive an active program of knowledge transfer about WHO recommendations for preeclampsia, including recommendations regarding antenatal corticosteroids for foetal benefits, antihypertensive therapy and magnesium sulphate for seizure prophylaxis. This study will have 90% power to detect a reduction in PTB associated with PE from a population estimate of 1.5 to 1.0%, representing a 33% risk reduction, and 80% power to detect a reduction from 2.0 to 1.5% (25% risk reduction). The necessary number of patients recruited to achieve these results is 750. Adverse events, serious adverse events, both anticipated and unanticipated will be recorded.

**Discussion:**

The PREPARE intervention expects to reduce PTB and improve care of women with PE without significant adverse side effects. If successful, this novel pathway of care is designed for rapid translation to healthcare throughout Brazil and may be transferrable to other low and middle income countries.

**Trial registration:**

ClinicalTrials.gov: NCT03073317.

**Electronic supplementary material:**

The online version of this article (10.1186/s12884-019-2445-x) contains supplementary material, which is available to authorized users.

## Background

Pre-eclampsia (PE) is a serious, placentally-mediated disorder that affects about 5% of pregnant women worldwide and represents a major cause of maternal and perinatal mortality [1, 2]. Additionally, the impact of PE on the immediate and long-term health of the infant cannot be overestimated. Apart from perinatal death, preeclampsia is associated with preterm birth (PTB) and fetal growth restriction, with important sequelae of chronic neurological disability and adult cardiometabolic diseases [3–5]. Although several of the pathophysiological features of PE (e.g. reduced placental perfusion, increased inflammation, oxidative and endoplasmic reticulum stress etc.) can contribute to these adverse outcomes, iatrogenic preterm birth persists as a major factor in many countries.

In Brazil, PE causes 23% of all maternal deaths. Once PE has been diagnosed, measures such as antihypertensive therapy and magnesium sulphate may reduce maternal morbidity (such as eclampsia or stroke), but the only ‘cure’ for PE is delivery, regardless of gestational age. For this reason, PE is the indication for almost 18% of all preterm births and nearly half of all iatrogenic premature deliveries [6–9]. In high-income countries (HIC), indicated deliveries for PE are an important contribution to PTB, but account for about 8% [4]. It is possible that many women with mild or equivocal diagnosis of PE may also be delivered inappropriately early in Brazil, often by Caesarean section, owing to imperfect diagnostic and risk stratification methods leading to hasty and unnecessary intervention.

It is evident that the decision for immediate or delayed delivery in women with preterm PE is complex. While immediate delivery serves the interests of the mother, delayed delivery enhances maturation of the fetus and improves neonatal survival rates [10]. Dr. Redman, one of the co-authors for this work, first developed methods in the United Kingdom more than 30 years ago for the expectant management of PE [11]. Considering the high rates of PTB related to PE in Brazil, a strategic approach for these cases becomes crucial. Therefore, this project aims to test whether the systemic application of an evidence-based risk stratification model can reduce preterm births among women with preterm PE in the country.

Two methods of risk stratification will be used to help obstetricians to make decisions regarding the best timing for delivery for women with suspected or diagnosed preterm PE. This application extends a recent Latin American trial of conservative management of very preterm PE, which did not have access to these objective risk stratifiers [12]. The first component of the PREPARE study intervention is the fullPIERS risk stratification algorithm. The fullPIERS algorithm is based on maternal symptoms, signs, and laboratory tests to accurately identify women at increased risk for maternal complications related to PE [13]. It predicts adverse maternal outcomes (AUROC 0.88) within 48 h but also performs well for up to the next seven days. Therefore, the fullPIERS model is able to identify those pregnant women who do not need immediate delivery, but do need continuing close surveillance. The second method uses the biomarker sFlt-1/PlGF ratio as a prognostic indicator for clinical need for delivery [14, 15]. In a prospective, multicentre, observational study, Zeisler et al. used a cohort of 500 women with suspected PE at less than 37 weeks to determine that sFlt-1/PlGF ratio cut-off of 38 had predictive value to indicate time to delivery. In this cohort, the negative predictive value of developing PE within one week was 98.9% with sensitivity of 88.2% and specificity of 80% [14]. In a validation cohort of additional 550 women, a cut-off of sFlt-1/PlGF ratio of 38 or lower had a negative predictive value of 99.3% for the development of PE within one week, sensitivity of 80% and specificity of 78.3%. Either fullPIERS or sFlt-1/PlGF strategies have more than 98% negative predictive value for adverse outcomes.

We will perform both of these determinations on women presenting with suspected or diagnosed PE in which hospital admission or delivery would usually be considered with conventional Brazilian practice. The fullPIERS assessment will be repeated twice weekly and sFlt-1/PlGF ratio weekly. For women with PE who are low risk by both assessments, we will recommend to care providers that delivery be delayed safely. Since greater than 95% of deliveries in Brazil occur in the hospital setting, this intervention is likely to have a high impact on reducing the number of unnecessary, iatrogenic preterm deliveries related to PE.

Changing the behaviour of care providers presents substantial challenges [16–22]. This is especially difficult in a setting in which we are changing what is likely perceived as a “less risky” to “more risky” approach with respect to maternal health. To address this challenge, a program of Knowledge Transfer (KT) will be introduced to all healthcare professionals involved with the management of pre-eclamptic patients in the participating centres [23, 24]. This program has been developed and tested by the Canadian Institutes of Health Research [25]. This KT will include information specifically relevant to the intervention. Thus it will include information about the excess of preterm births for precclampsia in Brazil, the hazards of preterm birth, the data upon which the intervention is based and the safety meausure instituted to assure the safety of the intervention.

In addtion, the PREPARE KT model will be based on the World Health Organization (WHO) recommendations for pre-eclampsia/eclampsia management, including the use of low-dose aspirin and calcium supplementation for prevention of pre-eclampsia. Evidence from meta-analyses indicates that calcium supplementation reduces the risk of pre-eclampsia by half (and even more in high-risk women). The benefit of aspirin is more modest with a 17% reduction in the risk of pre-eclampsia (25% in high-risk women) [3]. In Brazil calcium supplementation is given to less than 10% of women in whom it is indicated [26]. A recent work demonstrated high prevalence of inadequate calcium in two cohorts of pregnant women evaluated in Brazil [27].

Educational modules specific to the application and use of fullPIERS and sFlt/PlGF in the context of the trial will also be provided prior to implementation of the risk assessment strategy at each participating centre.

The PREPARE study will be implemented following a stepped wedge design. Clusters (in this case tertiary hospitals with maternity units) are identified to take part in a one-way cross-over cluster RCT and, before the trial begins, randomly allocated a start date from when they are given the intervention. The time they receive the intervention is randomly ordered [28]. The clusters crossover at regular intervals (in this case, every four months), from their current standard of care to the intervention program. The randomisation sequence is decided before the start of the trial, and is made known to each centre shortly before implementation.

This methodology is essential for this study because it would be challenging for one hospital to manage critical patients using two protocols concurrently, or to abandon the new, better methods when they have been introduced. Once the intervention has been introduced, it becomes part of the routine care for women with known or suspected preeclampsia in the hospital. Consent is carried out as for other routine hospital procedures.

The initial training in the new KT will be carried out by a skilled and experienced team moving from hospital to hospital. Also there will be a link to access e-modules for training that will be available for all professionals in the centres.

### Aims and hypotheses

The aims of the PREPARE study are:

- To test the hypothesis that risk stratification of women with suspected or confirmed pre-eclampsia based upon objective criteria reduces the proportion of medically-indicated preterm deliveries and improves neonatal outcome
- To evaluate whether use of and adherence to a Knowledge Transfer system will help to reduce preterm births in preeclampsia
- Collect samples and individual patient data to establish a database and biological sample bank to search for unique risks and pathophysiological features. This will facilitate future studies and allow direct comparisons of demographic, metabolic and genetic factors in Brazil, other LMIC (Low and medium-income countries) and in HIC

## Methods/design

This is a stepped wedge cluster randomised trial for the implementation of an intervention at hospital level to evaluate whether a risk stratification model can reduce the incidence of preterm deliveries among women with suspected or confirmed preeclampsia. The trial will be conducted in seven tertiary centres, geographically dispersed throughout Brazil, and will run for 48 months; with data collection for 32 months. The participating tertiary centres and respective cities are: Fernandes Figueira Institute, Rio de Janeiro; Maternity Maria Amélia Buarque de Holanda, Rio de Janeiro (these two hospitals constitute one centre for the study); Maternity Leila Diniz, Rio de Janeiro; Botucatu Medical School, Botucatu-SP; Center for Women’s Health – CAISM, Campinas-SP; Hospital Guilherme Álvaro, Santos-SP; Maternity Leonor Mendes de Barros, São Paulo; Hospital of Clinics of Porto Alegre-RS.

### Inclusion criteria

Women presenting with suspected or confirmed pre-eclampsia between 20 + 0 and 36 + 6 weeks’ gestation at any of the seven tertiary centres will be eligible. Women with any co-morbidity (e.g. chronic hypertension, renal disease, diabetes) will also be included. Pre-eclampsia will be defined as blood pressure (BP) ≥ 140 mmHg systolic or 90 mmHg diastolic on or after 20 weeks of pregnancy, plus proteinuria: more than 1+ on a qualitative dipstick, or ≥ 0.3 g/24 h or urine protein/creatinine > 30 mg/mmol. In the absence of proteinuria pre-eclampsia will be diagnosed if BP ≥ 140/90 mmHg in the presence of any relevant maternal symptoms, signs (e.g., severe hypertension), abnormal maternal laboratory criteria (e.g., low platelets), or abnormal fetal wellbeing (e.g., fetal growth restriction) [29].

### Exclusion criteria

Women presenting with diagnosis of fetal demise at admission, non-reassuring fetal status necessitating urgent delivery at admission, eclampsia, HELLP syndrome, renal, cardiac or respiratory failure, coma, active labour, abruption or emergent delivery for other indications will not be included in the risk stratification component of the study. Although these women will not be managed according to risk assessments, their data will be collected to understand the magnitude of the problem of uncontrollable emergencies related to preterm PE. In view of the complexities of preterm delivery with multifetal gestations independent of PE and the lack of established cut off values for the intervention proposed, this group of women will not be included. We will however collect data from this group for sensitivity analysis with the potential for later analysis.

### Randomisation, recruitment and collection of baseline data

Randomisation will be undertaken by the trial statistician through a computer-generated blocking list at the start of the trial, but the timing of implementation of the intervention to each centre will be revealed only 30 days before the transition phase.

**Fig. 1:**
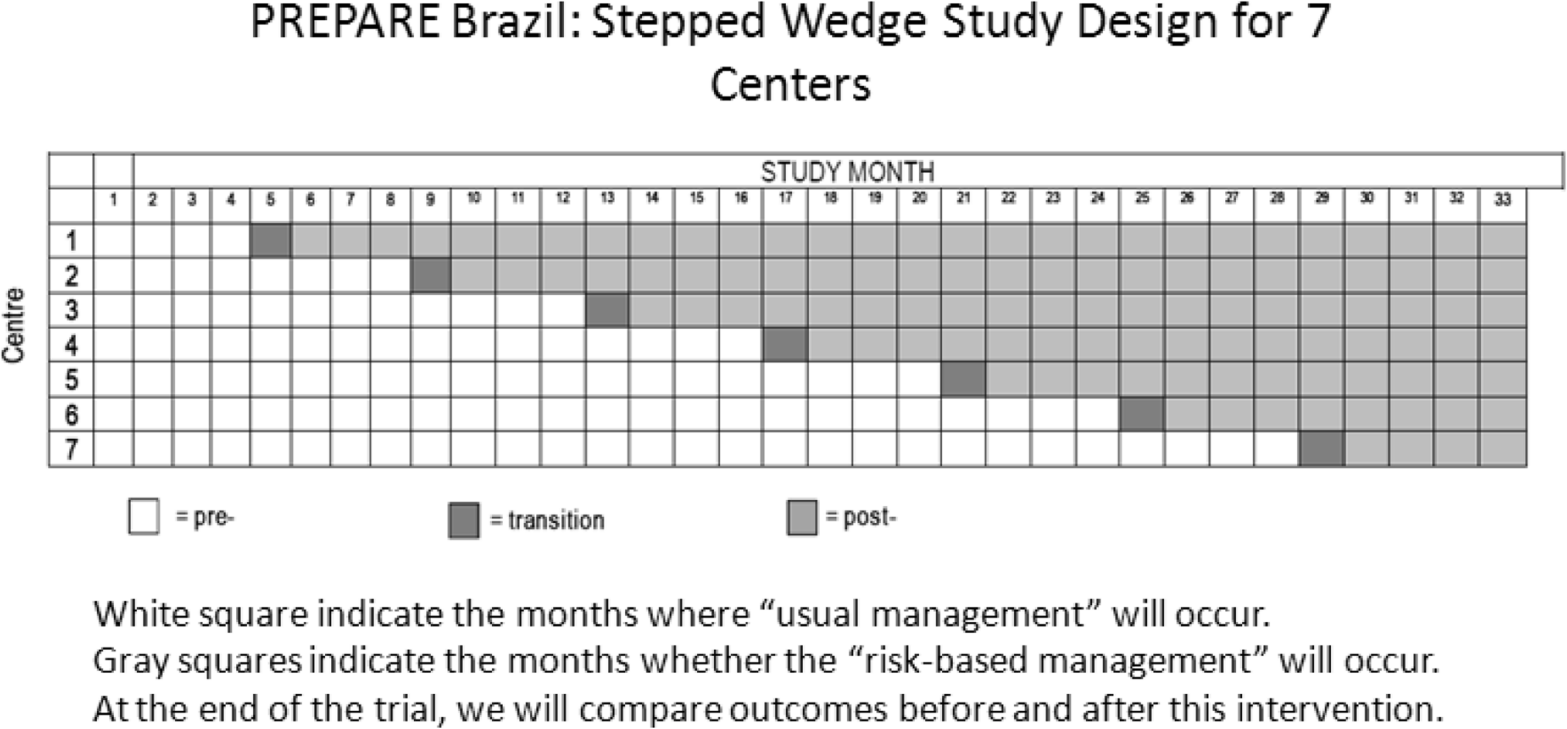
Description of randomisation and implementation of the intervention for the Stepped Wedge design. There will be an initial four-month period of baseline data collection at hospitals, during which none of the centres will be exposed to the intervention. Subsequently, every four months, one centre will be randomised to the intervention. Four months prior to implementation, the centre will be engaged in a programme of intensive knowledge transfer, followed by one month of transition

Recruitment will be performed by responsible doctors at each centre. At the start of the trial period, all women with suspected or diagnosed pre-eclampsia will be managed according to current local protocols. Subsequently, every four months, one centre will enter the transition phase to the implementation of the intervention (Fig. 1). All centres will continue the baseline data collection until randomised to implementation. Once each centre has crossed over to the intervention, it will continue to implement the intervention as part of routine care until the end of the study. After the last centre has crossed over, the trial continues a final four-month period during which all centres will be implementing the intervention. The effectiveness of the intervention will be measured by comparing the aggregated data of the centres in the pre-implementation phase of the trial with those in the post-implementation phase.

Routine clinical data will be collected for the trial on all pregnant women with suspected or diagnosed pre-eclampsia that present to the participating centres between 20 + 0 and 36 + 6 weeks’ gestation.

In addition, they will be invited to sign the informed consent form, and provide additional samples for research purposes.

### Intervention

Once the centre is randomised to receive the intervention, all eligible women, with suspected or diagnosed pre-eclampsia, will have sFlt-1/PlGF measurement and fullPIERS assessment performed as part of their routine care, and both results will be revealed to the care providers. sFlt-1 and PlGF will be measured using the Elecsys® Preeclampsia Platform (Roche Diagnostics) and the results will be entered onto the trial-specific MedSciNet database. Risk stratification using fullPIERS will be performed directly using a pre-defined risk scoring system integrated within the MedSciNet database. These results will be available within an amount of time (3–4 h), which permits for timely clinical decision-making.

**Fig. 2:**
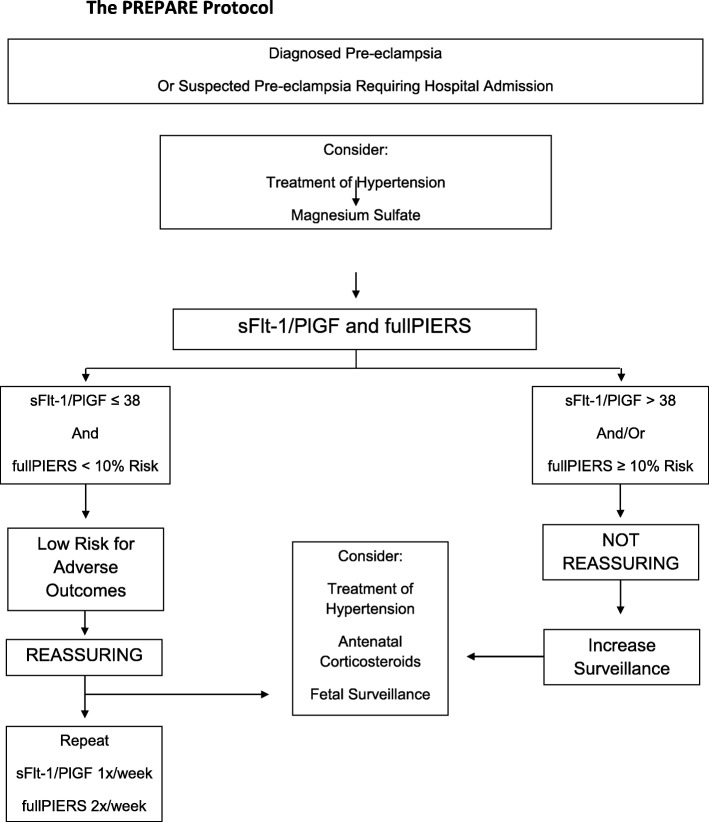
The PREPARE protocol for the intervention proposed. The care providers of women, who are low risk by both sFlt-1/PlGF and fullPIERS, will be informed of the woman’s risk status in 3–4 h, with a recommendation that delivery be delayed unless the clinical condition deteriorates. Women will be reassessed at least twice weekly with fullPIERS and at least weekly with sFlt-1/PlGF

The results will stratify eligible pregnant women into low-risk or not low-risk for adverse outcomes according to combined assessments of sFlt-1/PlGF and fullPIERS (Fig. 2). Low risk will be considered when sFlt-1/PlGF ≤38 and fullPIERS < 10% risk. The output provided by the platform will be: “Reassuring - low risk for adverse outcomes”, with recommendations that delivery be delayed unless the clinical condition deteriorates. Testing should be repeated at least twice weekly with fullPIERS and at least weekly with sFlt-1/PlGF for reassessment*.* The output for women stratified as not low risk (sFlt-1/PlGF> 38 and/or fullPIERS ≥10% risk) will be: Non-reassuring, with recommendations to increase surveillance based on WHO guidelines about delivery, including the need for preventative therapy with corticosteroids for fetal benefits, antihypertensive therapy and magnesium sulphate for seizure prophylaxis [3]. It is important to emphasize that the PREPARE protocol does not recommend delivery based on sFlt-1/PlGF> 38 and/or fullPIERS ≥10% risk. In these situations the PREPARE protocol recommends increased surveillance while delivery indications are based to local protocols and WHO criteria. Active knowledge transfer is a crucial part of this study and it will be directed primarily to clinicians, obstetric nurses, and technical staff.

### Outcome measures

#### Primary

Proportion of women delivered in the centres with preterm pre-eclampsia (deliveries with preterm pre-eclampsia/ total deliveries), where preterm is < 37 weeks’ gestation.

#### Secondary

Secondary outcomes will be:

- the proportion of women delivered with preterm pre-eclampsia out of all deliveries for pre-eclampsia;
- prolongation of pregnancy,
- proportion of women delivered < 34 weeks’ gestation with pre-eclampsia/total deliveries < 34 weeks’ gestation.

Additional maternal outcomes including

- maternal mortality (death in pregnancy or within 42 days of delivery)
- severe hypertension (systolic BP ≥ 160 mmHg or diastolic BP ≥ 110 mm/Hg),
- HELLP syndrome,
- pulmonary edema,
- eclampsia,
- stroke,
- coma,
- renal or hepatic dysfunction or thrombocytopenia;
- length of maternal hospital stay

Additional perinatal outcomes will include

- fetal death > 20 weeks’ gestation,
- perinatal death (stillbirth after 27^+ 6^ weeks or early neonatal death < 7 days of birth);
- Apgar score < 3 at 5 min,
- respiratory distress syndrome,
- necrotizing enterocolitis,
- confirmed sepsis,
- non-lethal seizures or coma,
- small for gestational age infants (< 10th and 3rd WHO centile),
- length of neonatal hospital stay.

### Knowledge transfer program

The activities for the Knowledge transfer (KT) program will be established in phases and constitute a strategy to improve commitment with recruitment and adherence to the study protocol.

Phase 1: the KT will address the problem of unnecessary delivery of women with preterm PE, its impact on fetal outcome and its magnitude in Brazil. The scale of and evidence for the problem will be presented to healthcare providers together with the proposed solution and its supporting information.

Phase 2: several strategies will be used of which the key component is the e-learning module [25]. One module will present the problem and supporting data and a second will outline the solution through the use of sFlt-1/PlGF and full-PIERS to grade the risks of delayed delivery. In both modules relevant data (including downloadable PDFs of an overview and/or the original manuscripts) will be available. All care providers in the participating centres will be expected to utilize this resource. Staffs who have omitted to use them will be prompted to do so. The final module will ‘test’ what has been learned and those who pass will be awarded a formal signed certificate of proficiency. The e-learning will be available to the providers at the time of the transition phase of the intervention. Seminars and meetings will complement these strategies.

Phase 3: the KT strategy for the study will be outlined and discussed. Participants will be asked to assess potential barriers, which can be minimised in future training interactions for Phase 4.

Phase 5: the use of the knowledge will be monitored and graded as monthly assessment during the intervention. Aspects regarding number of preterm births, whether all women for whom the intervention was indicated were treated and whether the information was used appropriately will be important points. If there is a suggestion that the intervention is not being performed when indicated or not used appropriately we will institute face-to-face seminars to identify and modify obstacles. The identification of specific practitioners who are accounting for the deficiencies we will meet with them for focussed determination of specific obstacles.

Phase 6: this phase will focus on clinical outcomes monitoring.

### Data management

All demographic and clinical data will be entered into a secure Internet based database designed and maintained by MedSciNetAB. This is the Global Pre-eclampsia Database which has been developed by CoLab (Global Pregnancy Collaboration) with MedSciNet and modified as appropriate for PREPARE. MedSciNet databases provide a secure and globally accessible data entry system that conforms to relevant FDA, NIH, and HL7 standards, guidelines, and recommendations (http://medscinet.com/projects.aspx). This data solution has been used for numerous clinical trials and studies globally, including several led by PIs of this project, and WHO studies in low to middle income countries. Data is held on software deployed on servers located in a data centre in Stockholm, operated in compliance with ISO27000 and SAS-70 standards and with 24 × 7 monitoring. Predefined reports are generated from an inventory, which logs sample deposition and removal.

Two research managers of the study will monitor data completion regularly and inform responsible researchers at each centre regarding problems to be addressed and corrected. All data must be completed up to 4 weeks after expected date to deliver.

Development of a biorrepository to test for unique pre-eclampsia features in Brazil, and for future mechanistic studies Additional file 2.

All women admitted with suspected or diagnosed pre-eclampsia will be invite to provide 20 mL of blood and 10 mL of urine. Therefore, plasma, serum, cells for DNA and urine will be stored in the facilities for long term sample storage (Biorepository). This biorrepository will be located at the Study Centre Laboratory, Medical School, Botucatu São Paulo State University, where -80 °C freezers with a nitrogen back-up system purchased for this purpose will be installed in the Laboratory for Clinical and Experimental Investigation with emergency power and alarms. In the event of freezer malfunction back-up freezers are available at this site for use during freezer repair. Informed consent will be requested for this purpose and will be taken by nurses that make part of the groups in each participant centre.

### Data safety and monitoring committee (DMC)

Monitoring of the accuracy and adequacy of data collection will be the responsibility of the Data Manager. He/she will investigate and wherever possible correct implausible entries and missing fields as they occur. Systematic problems will be referred to the Management Committee. A Data Monitoring Committee that will meet every four months will review the progress of the trial in terms of outcomes and results.

All responsible researchers by each centre have been instructed to report specific and important adverse outcomes involved with women participating in the trial. These specific adverse outcomes are: maternal death, fetal or neonatal death, eclampsia, HELLP syndrome, stroke and pulmonary edema requiring ventilation.

Rules for stopping the trial: It is possible for the intervention to result in inappropriate prolongation of pregnancy and serious events leading to adverse maternal or foetal/neonatal outcomes. The DMC will use the Haybittle-Peto rule, and stop the intervention if the *p*-value of the interim analysis is less than *P* < 0.001, and carry out a full interim analysis adjusting for baseline.

If these committees determine that this intervention is being used appropriately and increases adverse outcomes, then the trial will be stopped.

### Ethical consideration and trial registration

This study was approved by the national central ethical committee on research involving Human Subjects (CAAE: 53092916.4.2008.5411) and by each local ethical committee of the participating centres. The trial was registered at the ClinicalTrials.gov: NCT03073317.

The PREPARE Trial is hospital wide intervention, so all women admitted with suspected or diagnosed pre-eclampsia will be managed under local protocols during the baseline phase and under the PREPARE protocol during the intervention phase. Therefore, informed consent will be obtained for sample collection destined to be used in ancillary studies. All informed consent will be taken by the team involved in the project at each centre Additional file 1.

Important modifications necessary to protocol will be previously informed by the PI and all centres will be visited in order to clarify any changes.

### Statistical issues

#### Sample size

The primary aim is to test whether care targeted at preventing unnecessary delivery of women with pre-eclampsia will reduce the proportion of women delivered in the centres with preterm pre-eclampsia (deliveries with preterm pre-eclampsia/total deliveries). The power calculation depends on estimates of the total number of births, current pre-eclampsia prevalence and proportion with preterm deliveries from the seven centres (which have already agreed to participate). We estimate the mean number of infants born in each month per centre to be 330, and that of these ~ 5% will be born to women with pre-eclampsia. The preterm rate among those women with pre-eclampsia is approximately 36%. Therefore each month, there will be the potential to enter 17 women with pre-eclampsia, of whom approximately 6 would deliver prematurely, and outcome data will be available for approximately 700 deliveries of women with preterm pre-eclampsia. There is limited information on likely intracluster correlation (ICC) for this outcome in this setting and so we have explored sensitivity to a range of values.

Power calculations for stepped wedge cluster trials were as described in Hussy and Hughs [18]. Assuming that seven centres take part and allowing for a one month transition phase, and that in each centre there are approximately 17 cases of pre-eclampsia and 330 deliveries per month (with outcome data available for all deliveries), this study will have 89.6% power to detect a reduction in preterm deliveries associated with pre-eclampsia in the study centres from a population estimate of 1.5 to 1.0%, representing a 33% risk reduction, and 79.6% power to detect a reduction from 2.0 to 1.5% (25% risk reduction). Although the ICC is included in the power calculation, the design was found to be insensitive to the ICC and sensitivity analyses showed that these calculations were robust to larger magnitudes of ICCs.

### Data analysis

Hospital baseline characteristics of pregnancy outcomes to be collected throughout the data retrieval period will include numbers of women developing pre-eclampsia, women with preterm pre-eclampsia, gestational age at delivery of all hospital deliveries, and maternal and neonatal inpatient nights in women with pre-eclampsia. These baseline characteristics will be compared between exposure and non-exposure to the intervention and will be summarised by their means and standard deviations, medians and inter-quartile ranges, or numbers and percentages as appropriate. Centres will be classified as being exposed to the intervention one month after the centre was randomised to the intervention, and outcomes during the transition months will not be included.

The primary goal of the study is to test whether a care pathway targeted at preventing unnecessary delivery of women with pre-eclampsia will reduce the proportion of women delivered in the centres with preterm pre-eclampsia (deliveries with preterm pre-eclampsia/total deliveries). In statistical terms this null-hypothesis (no difference) can be tested using a mixed effects logistic regression model, with an indicator of whether the centre was exposed to the intervention as the dependent variable. Important independent variables to consider are the clustering effect (i.e. effect of centre), calendar time effect (since the intervention is sequentially rolled-out) and an indicator of intervention exposure for each centre at each time point; in additional to adjustment for other characteristics. Both individual and cluster level covariates to be included in the adjustment will be pre-specified and will include maternal age, parity, pre-existing chronic hypertension or other risk factors for prematurity. Null hypotheses and analyses for secondary outcomes take a similar form to that for the primary outcome, with appropriate link functions in the generalized linear mixed model. Summary treatment effect estimates will be reported (odds ratios or risk ratios) along with 95% CIs. These models will be fitted using population-averaged models using mixed effects methods in Stata version 14.2 or later (StataCorp, College Station, Texas).

The final trial dataset access will be limited to the authors of this study protocol.

Data will be disseminated through scientific meetings and peer-reviewed publications. Authorship of all publications will be shared among authors of this manuscript. Collaborators will be also listed in authorship as PREPARE research group.

## Discussion

Pre-eclampsia is heterogeneous with an unknown number of variants [1, 2]. We believe that there may be pathophysiological differences and other risks specifically relevant to Brazil and perhaps low-income countries in general. Therefore, the PREPARE study will bring the possibility of a better understanding of PE in these settings.

Remarkably, all information available on risk stratification in preeclampsia comes from high-income countries. Few studies have been done in the geographical settings where 99% of maternal mortalities occur. A national survey conducted in Brazil demonstrated a maternal near miss incidence of 10.2 per 1000 live births. Hypertensive disorders comprised the most prevalent complications in this group of patients [8]. Additionally, The Brazilian Network for Surveillance of Severe Maternal Morbidity (SMM), a cross-sectional multicentre study conducted in 27 referral hospitals across the country, classified 10 of these hospitals as providing inadequate care [30].

The surveillance of pre-eclamptic patients developed in PREPARE has the potential to qualify the health care assistance in the centres and if the intervention is implemented appropriately has the potential to reduce PTB and improve care of women with PE without significant adverse side effects. If successful, this novel pathway of care will be presented to health authorities to be adopted as part of a public health policy to help reduce SMM and maternal mortality rates in Brazil. This approach should be transferrable to other low or middle income countries or high-income settings with an excess of premature deliveries for preeclampsia.

## Additional files

Additional file 1: Informed consent for women admitted to hospitals with suspected or dignosed preeclampsia. (DOCX 15 kb)

Additional file 2: Declaration for sample use - PREPARE Biorrepository. (DOCX 13 kb)

## Data Availability

Not applicable.

## Abbreviations

BP: Blood pressure
DMC: Data safety and monitoring committee
fullPIERS: Model for risk stratification in preeclampsia
HELLP: Hemolysis, Elevated Liver, Low Platelets
HIC: High-income countries
KT: Knowledge transfer
PE: Pre-eclampsia
PlGF: Placental growth fator
PREPARE: Prematurity REduction by Pre-eclampsia cARE
PTB: Preterm birth
sFlt-1: Soluble fms-like tyrosine Kinase-1
SMM: Severe Maternal Morbidity
