# Supplementary material for: PREPARE: protocol for a stepped wedge trial to evaluate whether a risk stratification model can reduce preterm deliveries among women with suspected or confirmed preterm pre-eclampsia
Source: BMC Pregnancy Childbirth. 2019 Oct 7;19:343. doi: 10.1186/s12884-019-2445-x (PMC6781345; doi:10.1186/s12884-019-2445-x)
Supplement: Supplementary file 1 — Informed consent for women admitted to hospitals with suspected or dignosed preeclampsia. (DOCX 15 kb) [file 12884_2019_2445_MOESM1_ESM.docx]

Appendice I: Informed consent for women admitted to hospitals with suspected or dignosed preeclampsia

Title: **PREPARE – Prematurity Reduction by Preeclampsia Care**

Researcher:

Contact: Tel: e-mail:

Institution:

Adress:

___________________________________________________ (Hospital n^o^ ______)

Dear Patient

You are being admitted to the maternity due to suspected or diagnosed preeclampsia, a health problem characterized by high blood pressure. Preeclampsia can affect either you or your baby.

So, we invite you to participate in the PREPARE project, a study that aims to understand better what happens with patients who develop preeclampsia.

Preeclampsia constitutes a frequent pregnancy complication in Brazil and represents an important cause of preterm deliveries. So, the objectives of the PREPARE study is to provide better care to preeclamptic women and reduce the risks for preterm delivery.

In order to achieve these objectives you will be evaluated frequently and your baby will be monitored according to the current protocol. Some lab tests will be performed periodically to monitor the disease progression and define risks. Other monitoring procedures will be scans and cardiotocography.

Your participation in the study also includes the agreement in giving blood and urine samples. In order to reduce discomfort, sample collections will be performed during the collection for routine tests.

If you agree, 20mL of blood and 10mL with be collected and stored for this study and for future projects with similar purposes.

A questionnaire will be applied to you by one of the members of our team and all data recorded may be also used for research.

Regarding future researches you can choose to be or not to be contacted always when your data records and/or samples are used. This decision is up to you.

During the PREPARE study you will receive all necessary information regarding the project and any interventions. Either yours or your baby identification will never appear. Your data will be managed researchers with a patient ID.

_________________________ _______________________

Pacient signature Researcher signature

The risks related to your participation in the study are those regarding blood collection. It can be painful and you can have a local bruise.

The benefits related to your participation in the study include the possibility of improving quality of care for preeclamptic women, including yourself.

Your participation in the PREPARE study is voluntary and you can quit your participation at any time without any disadvantage for your treatment or for your baby.

The researcher responsible for the project can also remove your participation in the study if he judges that it brings any benefit to you or your baby.

All results found in all researches involving your participation may be published in scientific journals.

In addition to your agreement in participating in the PREPARE study you will be also asked to sign another form to demonstrate that you want or do not want to be contacted when your samples and data are used for future projects.

Your participation in the PREPARE do not involve any costs for you and you will not receive any payment. Compensations are guaranteed if you have any harm related to the research.

Additional information can be achieved at Ethics Committee on: ,directly with the researcher.

1 – Agreement.

“I, _________________________________________________________________

agree in participating in the research. I declare that I read and understood all contents of this document.

Signature____________________________________________________________

Date___/___/_______Telefone ___________________________________________

2 – Witness.

Name________________________________________________________________

Document_____________________________________________________________

Address/telefone________________________________________________________

Date___/___/_______ Signature___________________________________________

3 – Researcher or member of the team that presented this inform consent

Name________________________________________________________________

Signature_____________________________________________________________
