# Supplementary material for: PREPARE: protocol for a stepped wedge trial to evaluate whether a risk stratification model can reduce preterm deliveries among women with suspected or confirmed preterm pre-eclampsia
Source: BMC Pregnancy Childbirth. 2019 Oct 7;19:343. doi: 10.1186/s12884-019-2445-x (PMC6781345; doi:10.1186/s12884-019-2445-x)
Supplement: Supplementary file 2 — Declaration for sample use - PREPARE Biorrepository. (DOCX 13 kb) [file 12884_2019_2445_MOESM2_ESM.docx]

Appendice II: Declaration for sample use - PREPARE Biorrepository

I, _______________________________________________________________

holder of ID number___________________ , declare that I do not need tobe contacted to give additional informed consente regarnding the use samples for ancillary projects linked to PREPARE project.

I also declare that samples can be discarded by researchers if necessary.

Signature

Researcher information:
